# Bibliometric Analysis on of the Impact of Screening to Minimize Maternal Mental Health on Neonatal Outcomes: A Systematic Review

**DOI:** 10.3390/jcm13196013

**Published:** 2024-10-09

**Authors:** Maria Tzitiridou-Chatzopoulou, Georgia Zournatzidou

**Affiliations:** 1 Midwifery Department, School of Healthcare Sciences, University of Western Macedonia, GR50 100 Kozani, Greece; mtzitiridou@uowm.gr; 2 Department of Business Administration, University of Western Macedonia, GR511 00 Grevena, Greece

**Keywords:** screening, maternal mental health, digital training, diagnosis, perinatal depression, bibliometric analysis

## Abstract

(1) **Background**: Prenatal depression, maternal anxiety, puerperal psychosis, and suicidal thoughts affect child welfare and development and maternal health and mortality. Women in low-income countries suffer maternal mental health issues in 25% of cases during pregnancy and 20% of cases thereafter. However, MMH screening, diagnosis, and reporting are lacking. The primary goals of the present study are twofold, as follows: firstly, to evaluate the importance of screening maternal mental health to alleviate perinatal depression and maternal anxiety, and, secondly, to analyze research patterns and propose novel approaches and procedures to bridge the current research gap and aid practitioners in enhancing the quality of care offered to women exhibiting symptoms of perinatal depression. (2) **Methods**: We conducted a bibliometric analysis to analyze the research topic, using the bibliometric tools Biblioshiny and VOSviewer, as well as the R statistical programming language. To accomplish our goal, we obtained a total of 243 documents from the Scopus and PubMed databases and conducted an analysis utilizing network, co-occurrence, and multiple correlation approaches. (3) **Results**: Most of the publications in the field were published between the years 2021 and 2024. The results of this study highlight the significance of shifting from conventional screening methods to digital ones for healthcare professionals to effectively manage the symptoms of maternal mental health associated with postpartum depression. Furthermore, the results of the present study suggest that digital screening can prevent maternal physical morbidity, contribute to psychosocial functioning, and enhance infant physical and cognitive health. (4) **Conclusions**: The research indicates that it is crucial to adopt and include a computerized screening practice to efficiently and immediately detect and clarify the signs of prenatal to neonatal depression. The introduction of digital screening has led to a decrease in scoring errors, an improvement in screening effectiveness, a decrease in administration times, the creation of clinical and patient reports, and the initiation of referrals for anxiety and depression therapy.

## 1. Introduction

Perinatal depression, which is also referred to as perinatal depression, is a condition that affects approximately one in seven individuals during the perinatal period. Most of these patients, over 75%, do not receive any treatment for prenatal depression [1,2,3]. Perinatal depression may manifest during or prior to pregnancy and the postpartum period. Untreated perinatal depression is linked to an elevated risk of suicide and has adverse effects on the perinatal individual, the embryo (including premature birth and low birth weight), and the child (including poor attachment that can disrupt neurodevelopment). Additionally, it may have adverse effects on relationships with family members and social companions [4,5]. To diagnose perinatal depression, it is necessary to observe the presence of a minimum of five symptoms, including a despondent mood and a lack of interest or delight. These symptoms must be distinct from the individual’s typical functioning and must endure for a minimum of two weeks [6]. Furthermore, clinicians should assess the frequency, severity, and duration of perinatal depression symptoms, as well as probe for suicidal ideation and prior suicide attempts; inquire about substance use, including alcohol and opioids; evaluate the impact of current medications that may contribute to, imitate, or exacerbate perinatal depression; and screen for indications of bipolar disorder [7,8]. Additionally, they should gather information about the individual’s personal and family psychiatric histories; consider conducting tests for specific medical conditions, such as anemia and thyroid dysfunction; and inquire about substance use [9,10].

However, the absence of comprehensive data on the progression of symptoms associated with perinatal depression or any form of depression during pregnancy or the postpartum period has significantly restricted research on prenatal depression. Consequently, the development of new screening methods is essential and can contribute to the improvement and facilitation of therapies. Nevertheless, the digitalization of screening has occurred in recent years because of the integration of emergent technologies [11,12,13,14]. For instance, the quantity of and technological advancements in mobile phones have experienced unprecedented global surges. Mobile phones are significantly less likely to disrupt natural social behaviors than laboratory monitoring equipment because of their pervasive use, which renders them less noticeable [15,16]. Mobile phone applications are an effective aid in the continuous and frequent surveillance of mood states in women who are at a high risk of postpartum depression due to their broad appeal and immediate accessibility [17]. When patients are queried in a textual or in-app format, they are more likely to provide sensitive information in a comfortable environment, such as their own residence [18,19]. A study that was recently conducted examined the utilization of mobile applications to assess prenatal mental health [14].

Nevertheless, the global academic community is still in the process of identifying the field of digital screening and its significance in the prevention of perinatal depression symptoms. Consequently, the objective of the present study is to emphasize the transition from the conventional to the digital screening process and its impact on the enhancement of therapies that address perinatal depression symptoms. The research topic was addressed through the application of a bibliometric analysis using R Studio (4.4.0) and the bibliometric tools VOSviewer (1.6.20) and Biblioshiny (4.1). For this investigation, we obtained 197 documents from the Scopus and PubMed databases and employed Bibliometrix to analyze them. The results demonstrate a robust correlation between digital mental health and the screening of perinatal depression symptoms. Additionally, the findings underscore the significance of digital screening in the prevention of perinatal depression symptoms in refugee women, who are already at risk because of the circumstances and situations that force them to flee.

## 2. Perinatal Mental Health and Screening in the Digital Age

Perinatal mental health and psychosocial screening include gathering a number of responses from women related to queries about their present and past emotional and social well-being [20]. The objective is to ascertain the risk factors, symptoms, and indicators that are linked to the emergence of or advancement in a mental health disorder [9,10]. This may be accomplished as part of the standard prenatal and postoperative care that midwives, obstetricians, and other healthcare professionals provide. A critical component of perinatal mental health screening is the identification of women who are susceptible and may require additional support, as well as a comprehensive evaluation of their mental health. The failure to identify perinatal mental health problems during routine prenatal examinations is frequently the result of insufficient monitoring [21].

Screening may be performed using a structured questionnaire that offers predefined response options, or it can include a discussion with a healthcare practitioner in which open-ended questions are posed and answered. Screening is often performed using conventional techniques such as pen and paper, as well as contemporary digital technologies like the iCOPE digital prenatal mental health screening platform. Healthcare experts believe validated mental health and psychosocial screening devices to be credible. They provide a systematic method for addressing sensitive topics with mothers and improve the capacity to identify and provide timely treatment. The Australian clinical practice guidelines for mental healthcare during the perinatal period advocate for the use of many screening tools that have been designed and validated. These gadgets are often provided to women throughout pregnancy and after giving delivery. Their objective is to discern several facets of mental health and susceptibility to psychosocial challenges, including the likelihood of experiencing depression, anxiety, and other variables that provide protection [9,22].

According to national standards, it is advised that all women have regular screenings for depression and psychosocial risk factors at least two times during pregnancy and two times within the first year after giving birth. Within various prenatal and postnatal mental health settings, women may either neglect to receive regular tests or undergo screenings on many occasions. A range of approaches are used to provide healthcare during and after pregnancy, taking into account the woman’s personal choices, medical needs, unique circumstances, and region of residency [2,23]. Local primary healthcare services, prenatal clinics, public hospitals, private hospitals, mother and child health services, and auxiliary home visits compose the settings. The way women are tested for perinatal mental health and psychosocial risk factors varies across various healthcare settings, regions, and states, despite the existence of national standards, in terms of approach, scheduling, and scope. Research suggests that certain demographics have historically been underrepresented in perinatal mental health screening. These women comprise First Nations women, women born outside of the country, solitary or separated women, private patients, and elderly mothers. These populations remain underrepresented, despite an increase in perinatal mental health screening over time.

Additionally, the utilization of screening technologies is contingent upon the precise metrics that are implemented. Most perinatal mental health screenings are conducted through clinical evaluations in clinics and home visits, or by evaluating validated measures using paper-based and pen-based assessments [24]. There are several structural obstacles that impede the screening process for prenatal mental health. These include insufficient resources, poor mental health education and training for midwives and obstetricians, time constraints, issues with patient-provider relations, and shortages of resources. However, introducing computerized mental health screening during pregnancy and after childbirth may successfully reduce errors in scoring, increase the number of referrals for treatment for mental health issues, and improve time management. The integration of digital health into global healthcare is becoming more prevalent. This integration facilitates the efficient transfer of health information between patients and healthcare professionals while also improving decision making via the use of integrated algorithms and local care pathways [25]. This systematic review provides a clear definition of digital screening as the use of dependable and precise screening instruments, such as mobile phones, tablets, laptops, or desktop computers, by women throughout pregnancy and the postpartum period via the usage of mobile applications or a web-based connection.

It is essential to acknowledge the challenges and limitations related to this issue before advancing, despite the significant benefits of digital screening in mitigating perinatal depression symptoms, which considerably affect neonatal health. The limits and problems include the lack of evidence-based standards, privacy issues, data governance challenges, and ethical dilemmas [26]. The sensitivity of health data is a major worry, since its digitization might lead to privacy issues. approval presents an extra ethical dilemma, since many users may not fully understand the conditions of the use agreement when they provide their approval. Moreover, there is a paucity of research about the effects of digital screening technologies on health outcomes, cost-effectiveness, and system efficiency. The effectiveness of telehealth platforms may also be affected by users’ socioeconomic levels and incomes. Mothers who move to low-income or rural areas may have difficulties in understanding and using digital health solutions due to their reduced health literacy [26,27,28,29].

At present, there are no exhaustive evaluations that investigate the utilization of digital screening for mental health during pregnancy and postpartum. Therefore, the objective of this study is to ascertain whether digital screenings for mental health during pregnancy and postpartum are more effective, acceptable, and feasible than conventional treatments. A reliable referral for further evaluation is provided by efficient screening, which identifies women who have a higher likelihood of concurrently having a mental health disorder or detects indicators of mental health conditions during pregnancy and postpartum. According to professional standards, anxiety and depression are the most prevalent mental health disorders during the perinatal period. This process typically entails the assessment of these criteria in real-world scenarios [5,30,31]. The following factors determine the level of feasibility: initiation of referrals for depression and anxiety treatments, production of personalized clinical and patient reports, utilization of accessible and user-friendly technology, improvement in screening capabilities, and minimization of scoring errors. Furthermore, the aim of this systematic review is to determine the factors that either facilitate or impede the implementation of digital screening for mental health during pregnancy and postpartum. Furthermore, it endeavors to provide recommendations for the most effective digital diagnostic methods for perinatal mental health [32].

## 3. Materials and Methods

The objective of this research was to identify areas of growth, deficiencies, and recurring themes by employing a bibliometric methodology to evaluate articles in a systematic manner. Bibliometric analysis can be employed to ascertain the status of research and to identify prominent academic journals, publishing houses, or authors in a specific field. By employing the bibliometric method to investigate digital screening toward the symptoms of perinatal depression, it is possible to enhance one’s comprehension and acquire a comprehensive understanding of the academic subject. The inquiry evaluates aggregated literature data by employing the Scopus and PubMed, databases. In recent years, quantitative and bibliometric methodologies have become increasingly prevalent for the assessment of research output quality. An exhaustive examination is necessary to ascertain the efficacy, accuracy, and consistency of an assessment method.

The data for the present analysis were obtained from both Scopus and PubMed in June 2024. Furthermore, only articles published in English have been considered for the study of the specified document type. To provide more accurate bibliometric analysis findings, data from a whole year were assessed, resulting in the selection of publications published between 2011 and 2024. A prestigious bibliographic database, Scopus was established in 2004. Abstracts and citations from esteemed scientific journals compose the compilation. The database is composed of 36,377 titles that were acquired from 11,678 publishers. This investigation concentrates on the following four critical concepts: anguish, digital training, nurses, and postpartum depression. Also, the database of PubMed facilitated the exploration and recovery of biomedical and life sciences literature in order to enhance health outcomes on global and individual levels. The PubMed database has about 37 million citations and abstracts of the biological literature. Table 1 provides a comprehensive and detailed explanation of the keyword search procedure.

**Figure 1:**
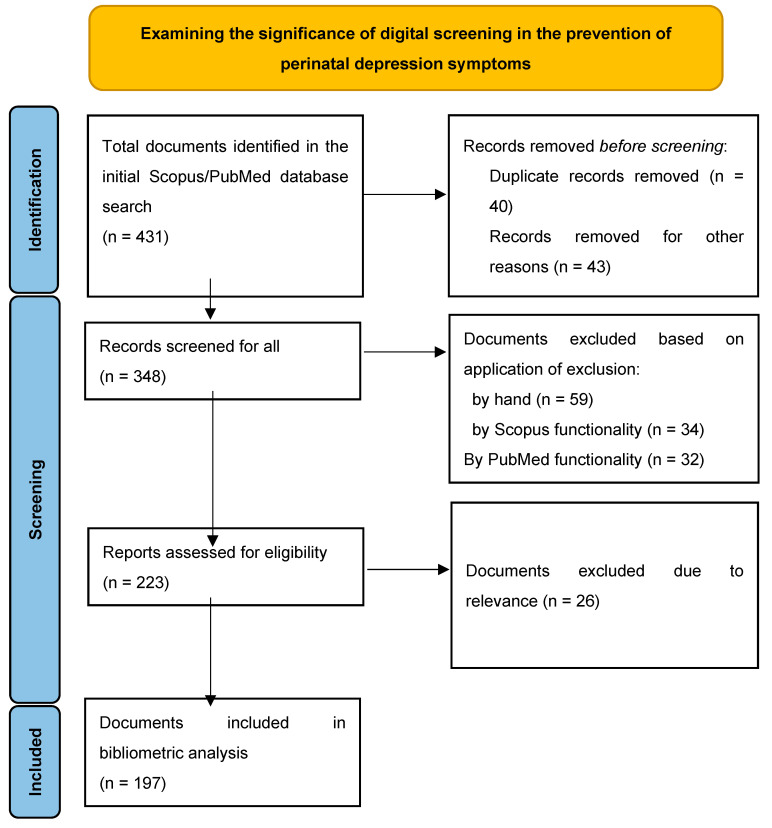
PRISMA flow diagram.

Furthermore, the PRISMA flow diagram visually illustrates the essential stages involved in choosing a dependable collection of publications for bibliometric analysis (Figure 1). The search query produced a grand total of 431 sources in the collection. Nevertheless, we limited the overall quantity of materials to 348 by only choosing periodicals. Following that, we carried out a comprehensive examination of a grand total of 223 papers, disregarding those that were too general or seemed unrelated to our current inquiry. The main objective of this inquiry is to highlight the significance of screening in reducing maternal and perinatal mental health symptoms, as well as the shift in screening methods to the digital age. Upon conducting a comprehensive analysis of the publications, we found that the title and keywords of numerous chosen sources did not explicitly mention the dimensions and qualities of the examined region. To exclude any extraneous references, we adjusted the search parameters to exclude articles that were not directly pertinent to the present study’s inquiry. A total of 197 scholarly articles were chosen and included in the bibliometric study after using this screening process.

## 4. Results

### 4.1. Content Analysis

**Figure 2:**
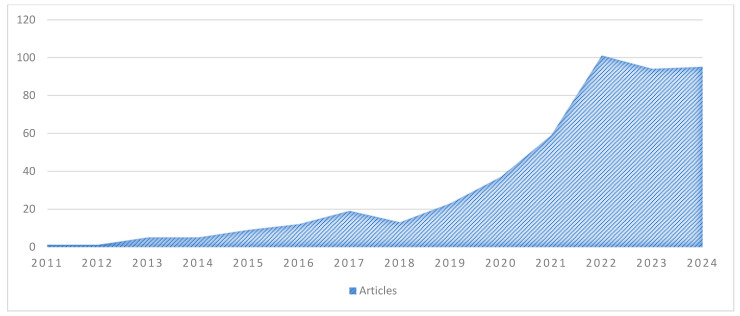
Annual production of scientific research in the field. Source: Scopus/Biblioshiny.

In recent years, the academic community has begun an investigation into the use of digital screening to prevent and mitigate the symptoms of maternal depression and its impact on the mental health of women and their infants. Figure 2 presents the scientific production in the field associated with the research topic spanning the years 2021 to 2024, as indicated by a query in the database of Scopus. The figure illustrates the exponential increase in the number of publications of this form of article over the past four years, culminating in a peak in the annual growth rate in 2022. The graph indicates a substantial increase in scientific production between 2021 and 2024, indicating an upward trend.

**Figure 3:**
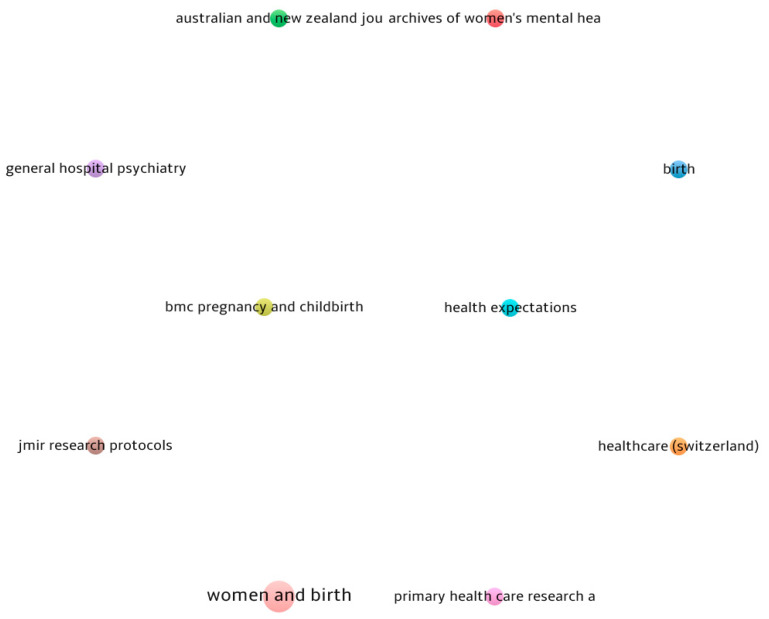
Most impactful sources in the field. Source: Scopus/VOSviewer.

Table 2 and Figure 3 both display the sources (journals) that received the most research submissions on the study topic between 2019 and 2023. *Women and Birth* is the source with the greatest number of relevant publications (45 documents) on the subject of detecting and mitigating symptoms of perinatal depression. Furthermore, the *Archives of Women’s Mental Health* ranked second among the sources for the greatest number of articles on the topic (34 documents). Additionally, the *Australian and New Zealand Journal of Psychiatry* is ranked third. This journal highlights the need of using digital screening to detect symptoms throughout the perinatal period, with a specific focus on refugee or migrant mothers.

**Figure 4:**
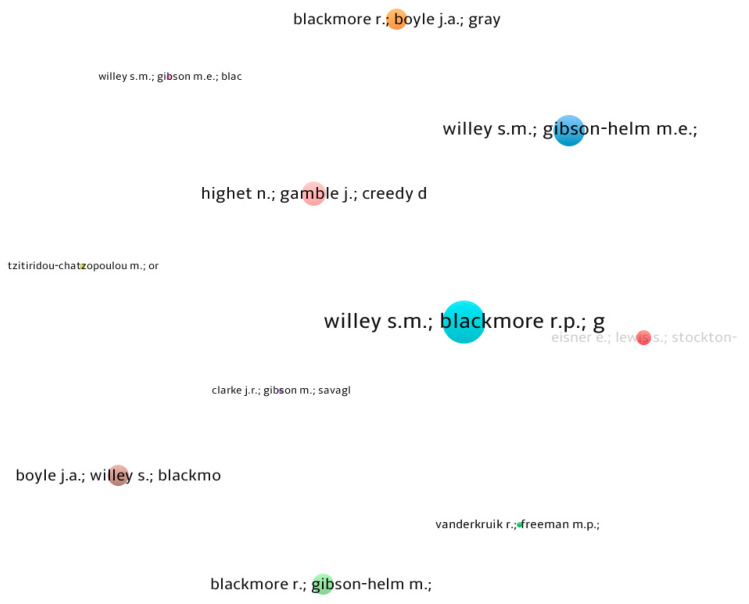
Most impactful documents in the field. Source: Scopus/VOSviewer.

Furthermore, Table 3 and Figure 4 provide a comprehensive analysis of the most significant articles on the subject, in addition to the bibliometric analysis. Research conducted by Willey et al. (2020), entitled “If you don’t ask, you don’t tell”: Refugee women’s attitudes on prenatal mental health screening”, aims to assess the feasibility and acceptability of a digital perinatal mental health screening program for women with a refugee background. The research’s findings highlighted the following three main topics: (i) women’s encounters with perinatal mental health screenings while pregnant; (ii) obstacles to and facilitators for obtaining continuous mental healthcare; and (iii) enhancements to the implemented screening programs, such as the creation of audio versions, which women found to be more practical and agreeable. In addition, the second study, entitled “Implementing innovative evidence-based perinatal mental health screening for women of refugee background”, agrees with the first paper. This is because the same authors who published the first study (Willey et al., 2019) authored this one as well, which may be seen as a preliminary version of the work entitled “If you don’t ask, you don’t tell: Refugee women’s perspectives on perinatal mental health screening”. A study entitled “Implementing innovative evidence-based perinatal mental health screening for women of refugee background” highlights the need of establishing national standards for regular screenings for depression and anxiety in all women throughout the perinatal period. The research contends that the effectiveness of regular pregnancy screenings is hindered by many obstacles at the service, community, and individual levels. Therefore, there is a pressing need to shift toward the utilization of digital screening methods [5,33].

**Figure 5:**
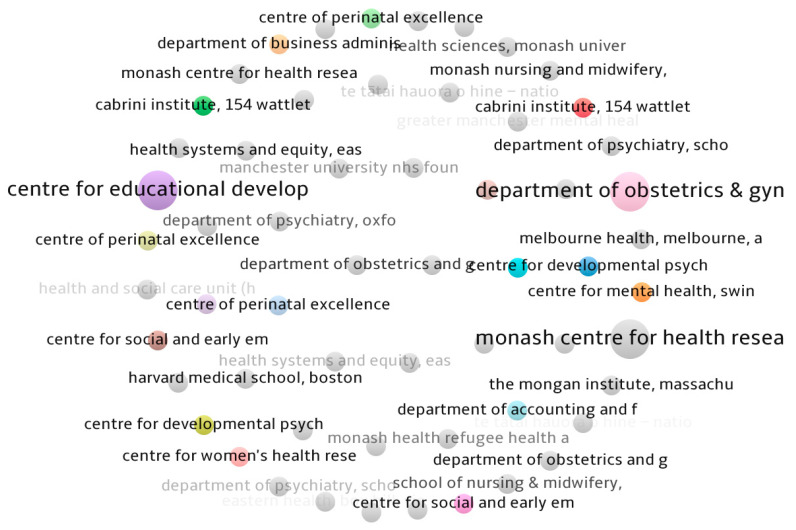
Most impactful affiliations in the field. Source: Scopus/VOSviewer.

Moreover, Figure 5 presents a map with the most impactful affiliations in the field. At the top of the list is the Department of Obstetrics & Gynecology at Monash University, which has an expertise on the screening technologies and outline the current evidence and best practice for managing the symptoms of perinatal depression. The Department of Obstetrics and Gynecology’s clinical research endeavors are integrated within the Ritchie Centre, a renowned university research facility and a leading perinatal and women’s health research cluster in Australia. The Ritchie Centre laboratories are located within Monash Health’s Translational Research Facility and are primary research collaborators with Monash Children’s Hospital and Monash Women’s Services. The Ritchie Centre’s objective is to promote the health of women and children by conducting innovative research that informs the development of more effective healthcare practices, such as digital screening.

### 4.2. Bibliometric Analysis

**Figure 6:**
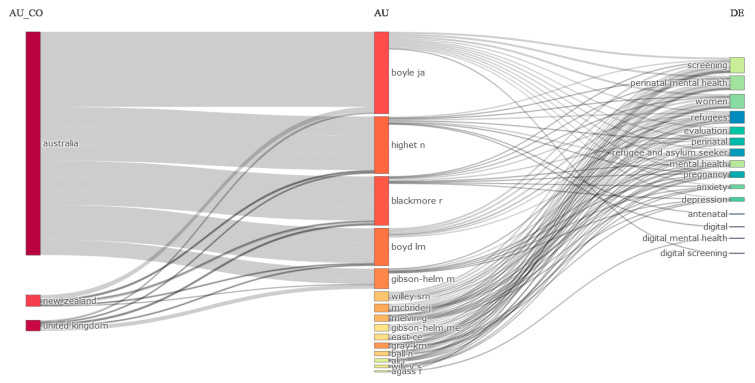
Three-field plot. Source: Scopus/PubMed/Biblioshiny.

Figure 6 presents the three-field plot, or Sankey diagram, which visualizes multiple attributes at the same time. Based on the findings from the plots, perinatal mental health may be improved by the use of digital technologies to connect screening and therapy. An analysis of the English-language research found that, on average, a mere 22% of women who test positive for perinatal depression actually receive treatment, despite the importance of screening for this disease. The discrepancy between screening and treatment may be worsened by the need for an extra 4 million behavioral health practitioners, as stated in a 2020 workforce study by the Substance Abuse and Mental Health Services Administration (SAMHSA). Increasing the availability of treatment to a larger number of women is crucial, since the shortage of providers and limited access to care pose substantial challenges [7,8]. Therefore, innovation is necessary to overcome these constraints. When used correctly, mental health apps may provide evidence-based therapies to women who are waiting for a healthcare professional, enhance traditional care to reduce the need for visits, or even substitute traditional care with peer support or psychoeducation.

Digital mental health solutions may also enhance accessibility for new moms. Amidst the pandemic, telemedicine has received favorable acknowledgment of its capacity to enhance accessibility for women in the postpartum period. These mothers may find that online medical treatment helps overcome challenges such as the need to arrange daycare or the hesitancy to bring young children to a medical facility. Considering that 85% of women own smartphones, the flexibility of apps to enhance care might make therapy more accessible compared to telemedicine alone. The ability to use an application at 4 AM while breastfeeding, instead of trying to rearrange a fragile sleep pattern to accommodate a doctor’s visit, may be a boon for busy new parents [36]. Additionally, around 50% of women have traumatic births [18], and the idea of revisiting a doctor’s office where the trauma occurred might be stressful. Remote treatment might allow individuals to assimilate the event at their own pace, rather than experiencing emotional distress or avoiding critical medical attention. Furthermore, alternative methods that are currently being developed have the potential to empower postpartum women to take control of their mental well-being, which is an essential aspect of trauma-informed therapy [37,38].

An analysis of evidence-based therapies for postpartum mental health may demonstrate how several modalities can facilitate the establishment of a customized care system for new mothers and their teams, according to their specific needs and availability. By incorporating traditional appointments, like synchronous and asynchronous support interventions, it may be possible to create a personalized treatment plan for each new mother instead of expecting patients to follow a predetermined care model.

**Figure 7:**
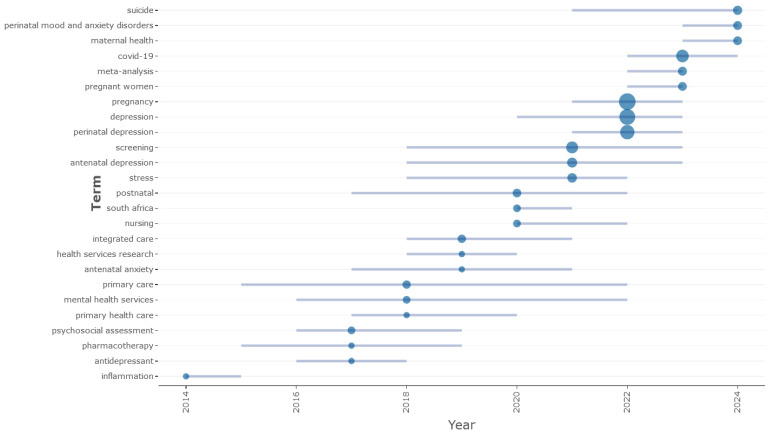
Research trends in the field. Source: Scopus/Biblioshiny.

Furthermore, Figure 7 highlights the research trends in the domain from 2011 to 2024. The figure illustrates the present and future directions of digital screening, highlighting its significance in preventing and alleviating maternal depression symptoms and its effects on the mental health of mothers and their children via co-occurrence network mappings. The keywords suggest that the pandemic epidemic activated several elements that affected women’s mental health. The myriad physical, emotional, and hormonal alterations linked to pregnancy and childbirth, alongside the forthcoming life transition and reconfiguration of the family unit, are significant factors contributing to the heightened vulnerability of women to depression and anxiety during and post-pregnancy. Nonetheless, the COVID-19 pandemic has had a significant influence. The quarantine, interruption of routine, and absence of social support have adversely affected new moms and their offspring. The confinement regulations significantly reduced the physical presence of the parental and social support network, which acts as a protective factor for mental health and, crucially, for suicide risk. The mental health of women, especially the most vulnerable, was significantly affected by the anxiety and worry stemming from the interplay of these causes and the widespread fear associated with the COVID-19 epidemic. Nevertheless, the data indicate that the screening approach for perinatal depression is clinically relevant, since any kind of sadness may profoundly affect a woman’s interactions with her spouse and baby after delivery.

### 4.3. Thematic Analysis

**Figure 8:**
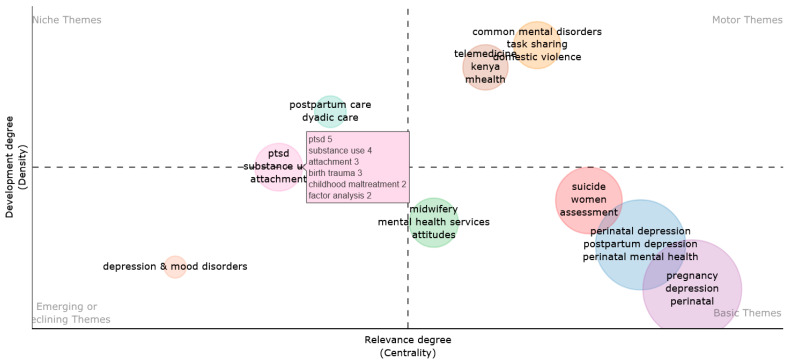
Thematic map. Source: Scopus/PubMed/Biblioshiny.

Furthermore, Figure 8 illustrates the research themes derived from the conceptual framework of the texts analyzed in the bibliometric study. The graph’s clusters represent the primary disciplines of study, and each cluster’s magnitude indicates the number of terms it encompasses. Each quadrant of the graph represents a distinctive concept. The image’s upper-right quadrant prominently displays motor theme-related motifs, showcasing the high concentration and compactness levels. The upper-left quadrant of the thematic map illustrates the addressed niche subjects. The group stands out because of its high density and low centrality. Additionally, the thematic map highlights the development of concepts in the lower-left quadrant, characterized by their limited prominence and concentration, while also highlighting significant topics in the lower-right quadrant. Their dominance in this field underscores the importance of telehealth and telemedicine in alleviating tension for both parents and infants during perinatal disorders.

The study’s thematic map reveals a significant association between the risk of posttraumatic stress disorder (PTSD), postpartum depression, and screening, all of which are distinct issues. Prenatal irritability significantly predicted heightened postpartum depressive symptoms, even when controlling for the following two robust predictors of postpartum depression: previous depression history and total trauma exposure. The research definitively indicates that a history of depression predicts postpartum depression; however, the relationship between trauma exposure and postpartum depressive symptoms is more intricate. The symptoms of PTSD, such as irritability, may function as critical indicators for assessment rather than only indicating the presence or absence of trauma. We should improve current screening methods for perinatal women by incorporating evaluations for both trauma and irritability to identify those at increased risk. We may modify depression screening instruments to include questions about irritability in addition to evaluating trauma history. A routine prenatal evaluation for trauma history and irritability may aid in identifying women at increased risk for postpartum depression before the likely onset of depressive symptoms.

The thematic mapping indicates that digital screening might be a very effective tool for aiding women in Africa in recognizing mental health issues associated with domestic abuse and pregnancy. During the perinatal period, symptoms of various mental illnesses, such as anxiety and depression, are notably widespread and associated with experiences of domestic abuse in Africa. Although research indicates that managing these symptoms throughout pregnancy improves health and economic outcomes for mothers and their children, pregnant women facing these challenges have limited access to regular screening and treatment. This is crucial for enhancing the welfare of perinatal moms and their children. The present theme analysis indicates that technology-based services are facilitating the alleviation of the mental health backlog for women suffering from pregnancy depression. Prior to the COVID-19 pandemic, pregnant women in need of mental health consultations were required to attend the hospital and arrange an appointment if necessary. Nonetheless, the revival of awareness has been necessitated by COVID-19. Although in-person consultations continue, the focus has shifted to enhancing access via digital screening techniques, since booking a virtual session has become quicker and more expedient than traditional methods.

**Figure 9:**
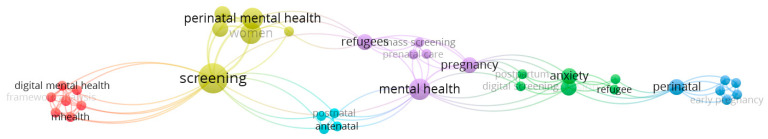
Keyword co-occurrence analysis. Source: Scopus/PubMed/VOSviewer.

Furthermore, Figure 9 illustrates that the VOSviewer application utilized a phrase co-occurrence analysis. Bibliometrix uses this form of analysis to highlight the importance of word co-occurrence clustering in digital screening, which helps to reduce symptoms of prenatal depression. In Figure 9, the font size and node area are determined by the weight value of the phrase. The frequency of the keyword’s occurrence is directly proportional to the weight value. The relevant node and font size grow in direct proportion to the weight value. If there is a line connecting two nodes, it indicates that the two terms are often used. The level of co-occurrence between the two words is shown by the thickness of the connecting line. The thickness of the connecting line is directly correlated with the degree of co-occurrence, meaning that a thicker connecting line represents a higher frequency of co-occurrence between the two phrases. In Figure 9, the analysis reveals the presence of six separate groupings.

However, the cluster that has the highest number of items, shown in red, is associated with the subject of digital mental health [13]. This emphasizes the significance of using digital tools, such cellphones, for the screening process. Furthermore, the relationship between the previously indicated cluster and the yellow cluster, which represents prenatal mental health and screening, is of similar significance. Furthermore, the co-occurrence study serves as a reminder of the need for the use of digital screening to detect indications of prenatal depression in refugees and migrants. Migrant and refugee women have similar risk factors for mental health problems as the local population during the perinatal period, such as isolation, financial difficulties, and physical health concerns [39,40]. However, migrant and refugee women face a range of distinct and interconnected risk factors, including uncertain immigration status, limited social support, and gender-based violence. In addition, female refugees or migrants face significant barriers when trying to access prenatal mental healthcare. Barriers to accessing healthcare include the intricate nature of the healthcare system, the high costs of treatments, and the lack of culturally and linguistically appropriate services, especially for women on temporary visas who are not eligible for Medicare. Government policies and services have not been adequately designed to address the perinatal support requirements of migrant and refugee women, nor have they focused on their preferred support interventions. This is despite the evidence indicating that migrant and refugee women face higher rates of perinatal mental health issues. At the policy level, there is a tendency to treat migrant and refugee groups as a homogeneous group, disregarding the distinct needs of people and communities, as well as the impact of gender on mental well-being.

The obstacles that hinder migrant and refugee women from getting help include the financial burden of services and the lack of gender-specific, culturally acceptable, or suitable perinatal mental health treatments at the organizational and sector levels. Therefore, it is crucial to provide prenatal mental healthcare that is specifically tailored to the gender, cultural background, and fair treatment of migrant and refugee women [23]. In order to meet the needs of migrant and refugee women throughout the perinatal periods, it is crucial to perform a comprehensive analysis that takes into account many elements and is based on evidence. This analysis should include the intersecting factors that contribute to the mental health risks faced by these women. The findings from this analysis will inform the development of policy changes. Hence, it is crucial to examine the capacity of digital screening to mitigate prenatal depression symptoms in this specific cohort of women, considering the circumstances and the susceptibility of refugee women [41].

## 5. Discussion

Symptoms of perinatal depression typically manifest within one to three weeks of the baby’s birth. Nevertheless, they may commence at any point during the initial year following the birth of the child. The symptoms are more severe than the infant blues and may encompass a profound pessimism and a complete lack of interest in the neonate. The health and development of the infant may be impacted by postpartum depression. Although it is not a common consequence of delivery, postpartum depression is a frequent occurrence. This may be the result of a variety of factors. Hormone levels may undergo abrupt fluctuations after pregnancy. Sleep deprivation, stress resulting from new regimens, and other changes may also induce postpartum depression. The prompt identification and treatment of postpartum depression are made possible by a postpartum depression screening. Additionally, the prevention of chronic depression may be facilitated by the early commencement of therapy. Medicine and therapy may be effective treatments for most individuals. In severe cases, treatment may involve brain stimulation techniques, including electroconvulsive therapy (ECT), which is occasionally referred to as “shock therapy”.

Nevertheless, the current research suggests that it is imperative to implement and integrate a computerized screening procedure to effectively and promptly identify and elucidate the symptoms of prenatal depression. The implementation of digital screening resulted in the reduction in scoring inaccuracies, an enhancement of screening efficiency, a reduction in the time required for administration, the generation of clinical and patient reports, and the commencement of referrals for anxiety and depression therapy. The selection of a user interface may influence the installation and adoption of digital screening. Nevertheless, these investigations were conducted on women who underwent screening at home at varying intervals, and it is uncertain whether these findings can be applied to clinical screening. The simplicity of instituting digital screening is facilitated by the information provided, capacity of the women to monitor their own actions and emotions, recommendation for social assistance, absence of scoring mistakes, and efficient self-completion. In general, women exhibited a high level of proficiency in the completion of digital screening, with minimal technical challenges. The screening was highly advantageous for them when it was conducted in their native language, as it was more efficient; they were able to comprehend the inquiries more readily, and they were more forthcoming with their responses. Digital screening has been demonstrated to effectively reduce humiliation, improve confidentiality, and promote equity among women and across various societies. Furthermore, women have demonstrated the ability to independently perform the operation in a sequestered clinic chamber by inputting their responses themselves using interactive voice response (IVR) technology.

The current research further demonstrates the need for digital screening in preventing prenatal depression symptoms in immigrant mothers [8,11,14,36]. Perinatal mental health concerns in migrant and refugee women are associated with social isolation, as well as a lack of adequate social support. Having a limited ability to speak English is a constant element that increases the likelihood of experiencing social isolation, especially for women who have moved as refugees or come from low-income and conflict-affected countries. The healthcare system may often be complex and difficult to navigate. Lack of culturally sensitive services and limited use of trained interpreters can create communication obstacles between healthcare providers and migrant and refugee women. These barriers can hinder the achievement of positive perinatal mental health outcomes and a safe pregnancy. Multiple studies have shown that the mental well-being of migrant and refugee women may be negatively impacted by health practitioners’ insufficient understanding of cultural beliefs and practices related to pregnancy and the period after giving birth [6,42,43]. Migrant and refugee women may feel excluded from healthcare choices made by healthcare professionals throughout their prenatal period since culturally appropriate health services are not readily available [9,10]. As a result, migrant women may have a reduced inclination to use perinatal mental health services and seek help for their emotional difficulties throughout pregnancy and postpartum. Therefore, digital screening will positively affect refugee women.

During pregnancy and after giving birth, digital screening provides a new, feasible, well-received, and efficient method for screening women for mental health problems, such as anxiety and depression [44,45]. Both women and healthcare professionals have demonstrated the feasibility and positive reception of this approach in clinical treatment. Key factors contributing to the success of this initiative include the availability of technological aid and support to help women understand the purpose and advantages of screening. Additionally, it is crucial to provide education and training to HCPs on screening, digital technology, and risk management for women. Digital screening empowers women to actively participate in their mental healthcare, referral, and treatment by enabling them to independently monitor and control their behavior [8,36]. Ensuring the availability of appropriate organizational resources and staff is crucial for promoting widespread usage, fairness, and availability of mental health assistance for women globally throughout the prenatal and postpartum period.

### Limitations

Certain limitations of the present study pertain to the use of bibliometric analysis. While bibliometric indicators may serve as a beneficial adjunct to the peer-review process, they are often misapplied and used without a comprehensive understanding of the underlying bibliometric research. Consequently, they are often used to assess metrics for which they were not designed or to draw comparisons that they are inherently incapable of facilitating.

## 6. Conclusions

The significance of digital screening in the prevention of prenatal depression symptoms is still being elucidated by the worldwide academic community. The objective of this inquiry is to highlight the importance of transitioning from conventional to digital screening processes and their impact on enhancing therapy for prenatal depression symptoms. The study topic was examined using bibliometric analysis with R Studio, together with the bibliometric applications VOSviewer and Biblioshiny. We used bibliometrix software to evaluate 197 papers acquired from the Scopus and PubMed databases for this study. The results demonstrate a significant association between the evaluation of prenatal depressive symptoms and digital mental health. Furthermore, the findings underscore the significance of digital screening in mitigating prenatal depression symptoms in refugee women, who are inherently vulnerable because of their precarious circumstances.

This research advocates for digital screening to mitigate prenatal depression among immigrant mothers. Social isolation and insufficient assistance are associated with prenatal mental health challenges in migrant and refugee women. Inadequate English proficiency increases the likelihood of social isolation, especially among women who are refugees or originate from low-income, conflict-affected nations. The healthcare system may be perplexing. In the absence of culturally relevant treatments and proficient interpreters, healthcare providers may have difficulties in communicating with migrant and refugee women. These impediments may hinder secure pregnancies and optimal perinatal health. Numerous studies have shown that health practitioners’ insufficient understanding of cultural attitudes and practices around pregnancy and postpartum may adversely affect the mental health of migrant and refugee women. Migrant and refugee women may feel marginalized in prenatal healthcare choices because of the scarcity of culturally appropriate health treatments. Migrant women may exhibit reduced likelihoods of using perinatal mental health treatments for emotional support throughout pregnancy and the postpartum period. Consequently, digital screening will assist refugee women.

This bibliometric review has identified key screening tools and practices in order to help healthcare providers mitigate symptoms of perinatal depression, which can affect mothers’ mental health and their neonates too, aid them in the implementation of digital screenings, and also provide recommendations for clinical practice. Future research and clinical practice should add to the literature by adapting current practices and implementing digital screenings for depression during pregnancy and postpartum in their specific healthcare settings worldwide, utilizing the theory-informed, best-practice recommendations presented in this systematic review, as well as various language translations and formats.

## Figures and Tables

**Table 1:** Keyword search formula.

Step	Keyword Search
1	((“screening” AND “perinatal depression”))
2	((“screening” OR “digital screening”) AND (“perinatal depression” OR “maternal perinatal depression”))
3	((“screening” OR “digital screening”) AND (“perinatal depression” OR “maternal perinatal depression”) AND (“maternal mental health”))
4	((“screening” OR “digital screening”) AND (“perinatal depression” OR “maternal perinatal depression”) AND (“maternal mental health” OR “maternal mental health disorders” OR “antenatal mental health”))
5	((“screening” OR “digital screening”) AND (“perinatal depression” OR “maternal perinatal depression”) AND (“maternal mental health” OR “maternal mental health disorders” OR “antenatal mental health”) AND “neonates”)
6	((“screening” OR “digital screening”) AND (“perinatal depression” OR “maternal perinatal depression”) AND (“maternal mental health” OR “maternal mental health disorders” OR “antenatal mental health”) AND (“neonates” OR “infants”))
7	((“screening” OR “digital screening”) AND (“perinatal depression” OR “maternal perinatal depression”) AND (“maternal mental health” OR “maternal mental health disorders” OR “antenatal mental health” OR “anxiety”) AND (“neonates” OR “infants”))
8	((“screening” OR “digital screening” OR “neonatal screening”) AND (“perinatal depression” OR “maternal perinatal depression”) AND (“maternal mental health” OR “maternal mental health disorders” OR “antenatal mental health” OR “anxiety”) AND (“neonates” OR “infants”))
9	((“screening” OR “digital screening” OR “neonatal screening”) AND (“perinatal depression” OR “maternal perinatal depression”) AND (“maternal mental health” OR “maternal mental health disorders” OR “antenatal mental health” OR “anxiety” OR “psychological risk”) AND (“neonates” OR “infants”))
10	((“screening” OR “digital screening” OR “neonatal screening”) AND (“perinatal depression” OR “maternal perinatal depression”) AND (“maternal mental health” OR “maternal mental health disorders” OR “antenatal mental health” OR “anxiety” OR “psychological risk”) AND (“neonates” OR “infants”)) AND (LIMIT-TO (DOCTYPE, “ar”)) AND (LIMIT-TO (PUBSTAGE, “final”) OR LIMIT-TO (PUBSTAGE, “aip”)) AND (LIMIT-TO (SRCTYPE, “j”))

**Table 2:** Most relevant sources. Source: Scopus/PubMed/Biblioshiny.

Sources	Articles	Subject Area
*Women and Birth*	45	Maternity and Midwifery
*Archives of Women’s Mental Health*	34	Obstetrics and Gynecology
*Obstetrics & Gynecology*	31	
*Birth*	24	Obstetrics and Gynecology
*Bmc Pregnancy and Childbirth*	19	Obstetrics and Gynecology
*JAMA Pediatrics*	17	
*Health Expectations*	10	Public Health, Environmental, and Occupational Health
*Healthcare* (Switzerland)	6	Health Policy
*Journal of Midwifery & Women’s Health*	6	
*Nursing for Women’s Health*	5	

**Table 3:** Most relevant documents in the field. Source: Scopus/Biblioshiny.

Paper	Total Citations	TC per Year
“If you don’t ask … you don’t tell”: Refugee women’s perspectives on perinatal mental health screening [33]	22	4.40
Implementing innovative evidence-based perinatal mental health screening for women of refugee background [5]	13	2.60
Perinatal mental health and psychosocial risk screening in a community maternal and child health setting: evaluation of a digital platform [23]	9	1.50
Introducing and integrating perinatal mental health screening: Development of an equity-informed evidence-based approach [9]	7	2.33
Improving Mental Health in Pregnancy for Refugee Women: Protocol for the Implementation and Evaluation of a Screening Program in Melbourne, Australia [34]	7	1.17
Validation of a Dari translation of the Edinburgh Postnatal Depression Scale among women of refugee background at a public antenatal clinic [35]	7	2.33
Digital screening for postnatal depression: mixed methods proof-of-concept study [14]	4	1.33
To screen or not to screen: Are we asking the right question? In response to considering de-implementation of universal perinatal depression screening [30]	1	0.50
Digital Training for Nurses and Midwives to Improve Treatment for Women with Postpartum Depression and Protect Neonates: A Dynamic Bibliometric Review Analysis [32]	0	0.00
Digital screening for mental health in pregnancy and postpartum: A systematic review [13]	0	0.00

## Data Availability

Dataset available upon request from the authors.
